# HSV-2 Specifically Down Regulates HLA-C Expression to Render HSV-2-Infected DCs Susceptible to NK Cell Killing

**DOI:** 10.1371/journal.ppat.1003226

**Published:** 2013-03-28

**Authors:** Moran Elboim, Inna Grodzovski, Esther Djian, Dana G. Wolf, Ofer Mandelboim

**Affiliations:** 1 The Lautenberg Center for General and Tumor Immunology, The BioMedical Research Institute Israel-Canada of the Faculty of Medicine, The Hebrew University Hadassah Medical School, Jerusalem, Israel; 2 Virology Unit, Hadassah Hospital, The Hebrew University Hadassah Medical School, Jerusalem, Israel; McMaster University, Canada

## Abstract

Both NK cells and CTLs kill virus-infected and tumor cells. However, the ways by which these killer cells recognize the infected or the tumorigenic cells are different, in fact almost opposite. CTLs are activated through the interaction of the TCR with MHC class I proteins. In contrast, NK cells are inhibited by MHC class I molecules. The inhibitory NK receptors recognize mainly MHC class I proteins and in this regard practically all of the HLA-C proteins are recognized by inhibitory NK cell receptors, while only certain HLA-A and HLA-B proteins interact with these receptors. Sophisticated viruses developed mechanisms to avoid the attack of both NK cells and CTLs through, for example, down regulation of HLA-A and HLA-B molecules to avoid CTL recognition, leaving HLA-C proteins on the cell surface to inhibit NK cell response. Here we provide the first example of a virus that through specific down regulation of HLA-C, harness the NK cells for its own benefit. We initially demonstrated that none of the tested HSV-2 derived microRNAs affect NK cell activity. Then we show that surprisingly upon HSV-2 infection, HLA-C proteins are specifically down regulated, rendering the infected cells susceptible to NK cell attack. We identified a motif in the tail of HLA-C that is responsible for the HSV-2-meduiated HLA-C down regulation and we show that the HLA-C down regulation is mediated by the viral protein ICP47. Finally we show that HLA-C proteins are down regulated from the surface of HSV-2 infected dendritic cells (DCs) and that this leads to the killing of DC by NK cells. Thus, we propose that HSV-2 had developed this unique and surprising NK cell-mediated killing strategy of infected DC to prevent the activation of the adaptive immunity.

## Author Summary

Approximately 20% of all humans are latently and asymptomatically infected with HSV-2. This suggests that the virus developed mechanisms to avoid immune cell detection; many of which are still unknown. Infected cells are killed mainly by two lymphocyte populations; NK cells and CTLs that belong to the innate and the adaptive immunity, respectively. While the killing machinery of these two cell types is similar, almost identical, the ways by which they discriminate between infected and uninfected cells is different. CTLs are activated, primarily by DCs, to become effector cells. They then recognize virus-derived peptides in the groove of MHC class I molecules and eliminate the virally infected cells. In contrast, NK cells recognize infected cells through several NK cell activating receptors, while the recognition of MHC class I proteins by NK cells leads to inhibition of NK cell killing. Viruses, such as HIV, developed mechanisms to interfere with the function of both NK cells and CTLs via targeting of specific MHC class I proteins. Here we show that HSV-2 developed a MHC class I-dependent mechanism in which the virus, through specific targeting of HLA-C by the viral protein ICP47, harness the NK cells for its own benefit, probably to avoid the activation of adaptive immune response.

## Introduction

Human Natural killer (NK) cells comprise approximately 5–15% of peripheral blood lymphocytes. They kill infected or transformed cells and can also contribute to the activation of the adaptive immunity through the secretion of cytokines and chemokines [1]. Additionally, NK cells regulates adaptive immunity through the killing of autologous immune cells including activated T cells and DCs [2]. They can also kill autologous self cells such as beta cells [3] and stellate cells [4]. The activity of NK cells is controlled by the balance of signals delivered by inhibitory and activating receptors [5], [6]. Thus, NK cells can be activated by induction in the expression of activating ligands and/or by reduction in the expression of inhibitory ligands [7]. A group of NK inhibitory receptors interacts specifically with MHC class I (MHC-I) proteins. These receptors prevent the NK cell-mediated attack of normal cells, whereas cells with compromised MHC-I expression become susceptible to NK cell-mediated killing [8]. The MHC-I molecules in humans comprise the classical HLAs: HLA-A, HLA-B and HLA-C, and the non-classical HLA-E, HLA-F and HLA-G molecules [9]. Practically all of the HLA-C alleles can be divided into two groups, in terms of NK cell recognition, based on the residue located at position 80 [10]. The HLA-C1 group, that includes for example HLA-Cw3 and HLA-Cw7, is characterized by the presence of asparagine in position 80 and is recognized by the KIR2DL2 receptor. The HLA-C2 group, which includes proteins such as HLA-Cw4 and HLA-Cw6, is characterized by the presence of lysine in position 80 and is recognized by the KIR2DL1 receptor [8], [10], [11]. Since, virtually all of the HLA-C molecules belong to either group 1 or group 2 it is thought that the HLA-C molecules were probably developed to primarily inhibit the NK cell activity. In marked contrast, cytotoxic T lymphocytes (CTLs) execute their cytolytic activity upon interaction with MHC-I proteins. Stable peptide/MHC-I complexes are assembled in the ER and transported to the cell surface where they are inspected by the T-cell receptors (TCR) of CTLs [12]. If the MHC-I proteins carry foreign peptides, derived from viral or tumor antigen, then the cells carrying these complexes will be killed by CTLs. Hence, MHC-I has a dual opposite role with regard to innate and adaptive immunity as on the one hand it inhibits NK cells activity and on the other hand it activates CTLs. Viruses in general and especially viruses that establish long-term infections in their hosts, have evolved a number of mechanisms to down regulate MHC-I expression [13]. However, a general down regulation of MHC-I proteins is problematic because although the infected cells will be protected from killing by CTLs, they will become more susceptible to NK cells attack. Therefore, some sophisticated viruses such as HIV developed mechanisms to specifically down regulate certain HLA alleles such as HLA-A and HLA-B, which are especially important for HLA-TCR interaction and leave on the cell surface the HLA-C proteins to inhibit the NK cell activity [14], [15]. Herpes simplex virus type 2 (HSV-2) is a double-stranded DNA virus, belonging to the Herpesviridae family. Its genome is very large (155 kb), encoding for at least 84 genes [16]. Each year around 500,000 people in the United States are infected with HSV-2, and at least 22% of the population has a latent infection [17]. NK cells play an important role in fighting HSV-2 infections. Lower rate of mice survival and higher viral titers were observed in the vaginal mucosa of IL-15^−/−^ deficient mice that lacks NK cells and in mice in which NK cells were depleted [18]. Moreover, NK cells were shown to be the main source of IFN-γ secreted in the mice genital tract in the first three days following HSV-2 infection [18], [19] and in agreement with this, IFN-γ deficient mice were more susceptible to HSV-2 infection than wild type mice [18]. Finally, it was shown recently that genital HSV-2 infection induces short-term NK cell memory [20]. Herpes viruses are well known for their sophisticated immune evasion mechanisms. However, not much is known about the immune evasion strategies of HSV-2. The best example with this regard is probably the HSV-2 ICP47 protein, a viral immediate early protein that blocks peptide binding to the transporter associated with antigen processing (TAP) and consequently reduces MHC-I expression [21]. The efficiency however of ICP47 is cell type dependent and in cells that express high levels of TAP (such as antigen-presenting cells (APC)) ICP47 poorly inhibits MHC-I antigen presentation [21], [22]. Here we describe a novel and unique immune evasion mechanism of HSV-2 that is specific to humans as we demonstrate that HSV-2, unlike any other virus known to date, specifically down regulates the expression of HLA-C. We identify the mechanism leading to this unexpected and surprising down regulation and we show that it leads to NK cell-mediated killing of HSV-2 infected DCs. We suggest that the virus had developed this unique strategy to interfere with activation of the adaptive immunity response.

## Results

### Enhanced NK Cell Killing Following HSV-2 Infection

**Figure 1:**
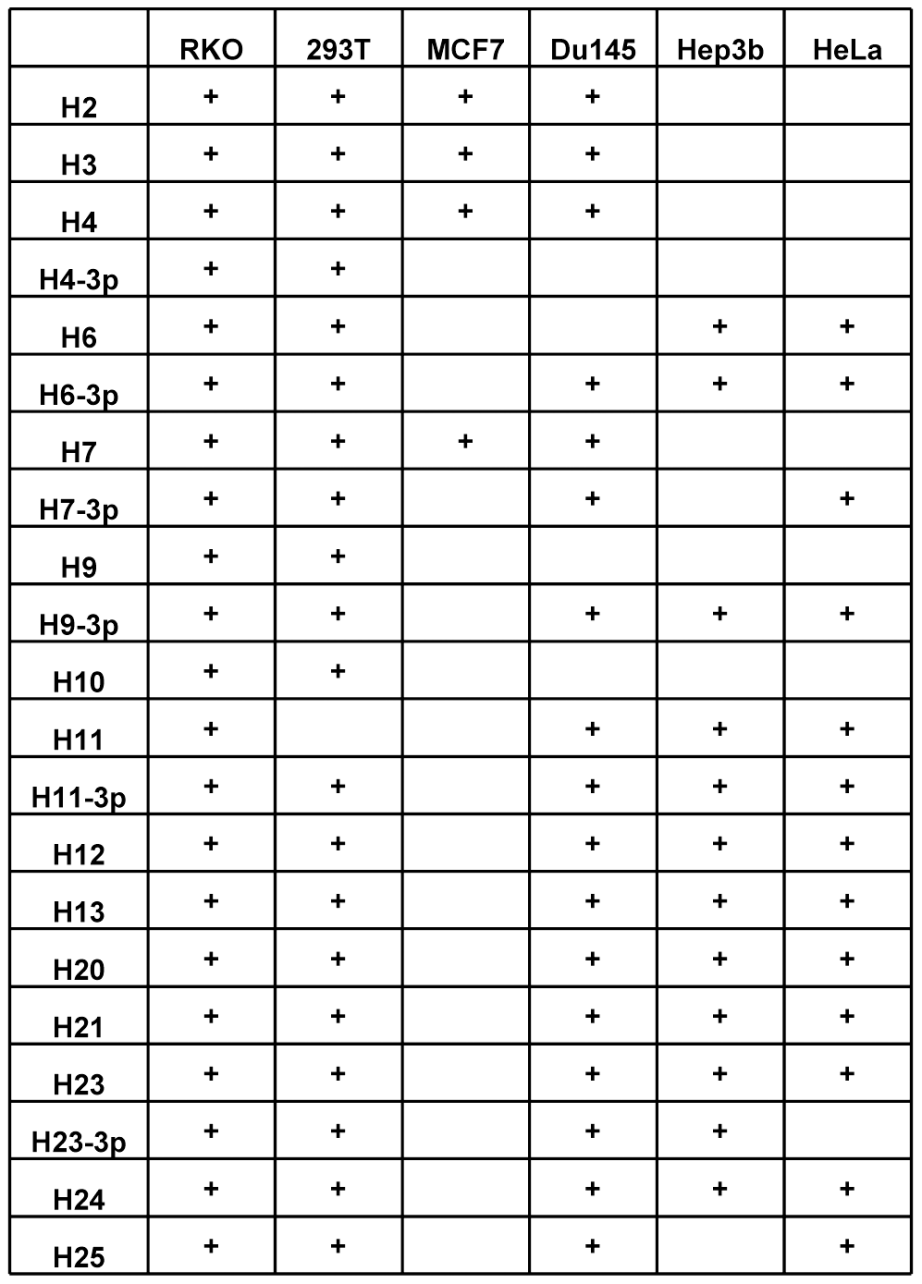
Various NK cell ligands are not downregulated by HSV-2 miRNA. Various human cell lines indicated in the table were transduced with lentiviruses expressing GFP together with various HSV-2 miRNAs or with control miRNA. Expression levels of MHC-I, MICA, MICB, ULBP1, ULBP2, ULBP3, PVR and ICAM1 were assessed by FACS. The figure displays the human cell lines that were transduced with each of the 21 miRNAs of HSV-2. Plus indicate that the cell line was tranduced with the indicated miRNA and tested for expression of the indicated NK ligands.

**Figure 2:**
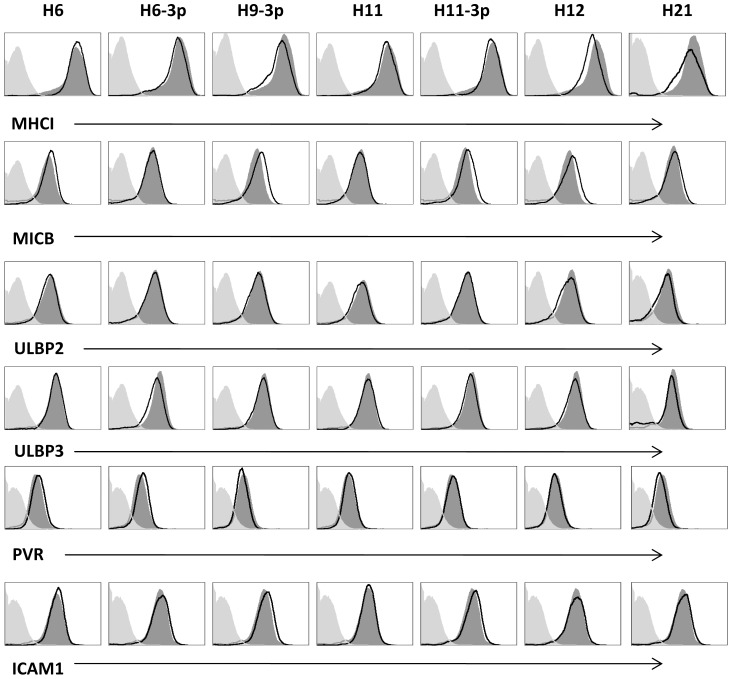
Selected examples of ligands for NK cell receptors that are not downregulated by HSV-2 miRNA. RKO cells were transduced with lentiviruses expressing GFP together with either HSV-2 miRNAs (black empty histogram, the miRNA are indicated on the top of the histograms) or with control miRNA (dark gray shaded histogram). Expression levels of MHC-I, MICB, ULBP2, ULBP3, PVR and ICAM1 were assessed by FACS using specific antibodies. Background levels (light grey shaded histogram) are the APC-conjugated Abs. The figure show one representative experiment out of three performed.

**Figure 3:**
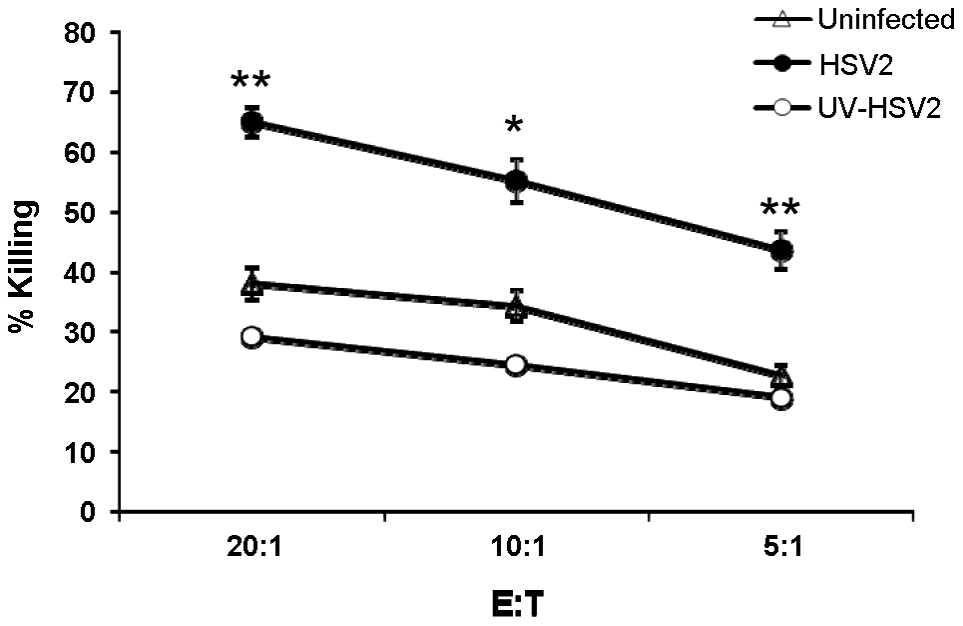
Increased NK cell killing of HSV-2 infected HFF cells. HFF cells, either uninfected, infected with HSV-2 (MOI of 0.5) or infected with UV-inactivated HSV-2 (MOI of 0.5) were radioactively labeled and incubated with primary bulk NK cell cultures for 5 hours at the indicated effector to target ratios (E:T). The killing assays were performed 48 hours post infection. Shown are mean values ± SD. Statistically significant differences are indicated (_*_ p≤0.05, _**_ p≤0.005, by one-tailed t test). Error bars (SD) are derived from triplicates. One representative experiment is shown out of three performed.

We have previously demonstrated that the miRNAs of several herpes viruses: HCMV, KSHV and EBV target the stress-induced ligands of NKG2D to avoid attack by NK cells [23], [24]. To test whether the miRNAs of HSV-2 are also involved in the regulation of NK cell killing we expressed 21 of HSV-2 microRNAs (miRNAs) in various human tumor cell lines that endogenously express NK cells ligands (Figure 1, summarizes the human cell lines that were transduced with each of the 21 miRNAs of HSV-2 and (Figure 2)). To our disappointment, none of the HSV-2 miRNAs affected the expression of MHC-I, MICA, MICB, ULBP1, ULBP2, ULBP3, PVR and ICAM (data not shown, Figure 1 and selected examples in Figure 2). To determine whether HSV-2 has developed other strategies to escape NK cell cytotoxicity we infected primary Human Foreskin Fibroblast (HFF) cells either with HSV-2 or with a UV-inactivated HSV-2 virus which is able to infect the cells but is unable to *de novo* produce viral gene transcripts. As can be seen in figure 3, HSV-2-infected HFF cells were killed more efficiently than the uninfected ones. Similar results were obtained at various time points post infection (data not shown). This enhanced killing was due to the production of viral gene products as infection with a UV-inactivated virus did not enhance the NK cell killing of HFF cells (Figure 3). Thus, surprisingly and in contrary of what we have expected, HSV-2 viral products are involved in enhancing NK cell cytotoxicity.

### Specific Down Regulation of HLA-C Following HSV-2 Infection

**Figure 4:**
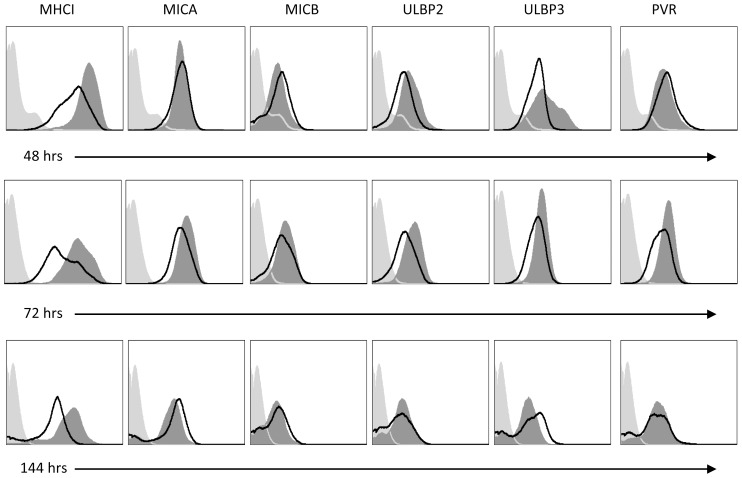
HSV-2 specifically downregulates MHC-I expression. Time course for the expression of various NK cell ligands following HSV-2 infection of HeLa cells (MOI of 0.1). The expression levels of NK cells ligands (indicated on top of the histograms) were determined by staining with specific antibodies. Expression following infection is indicated by the black empty histograms. The dark gray shaded histograms represent staining of the corresponding uninfected cells. Background levels (light grey shaded histogram) are the secondary FITC-conjugated Abs staining. One representative experiment is shown out of three performed.

**Figure 5:**
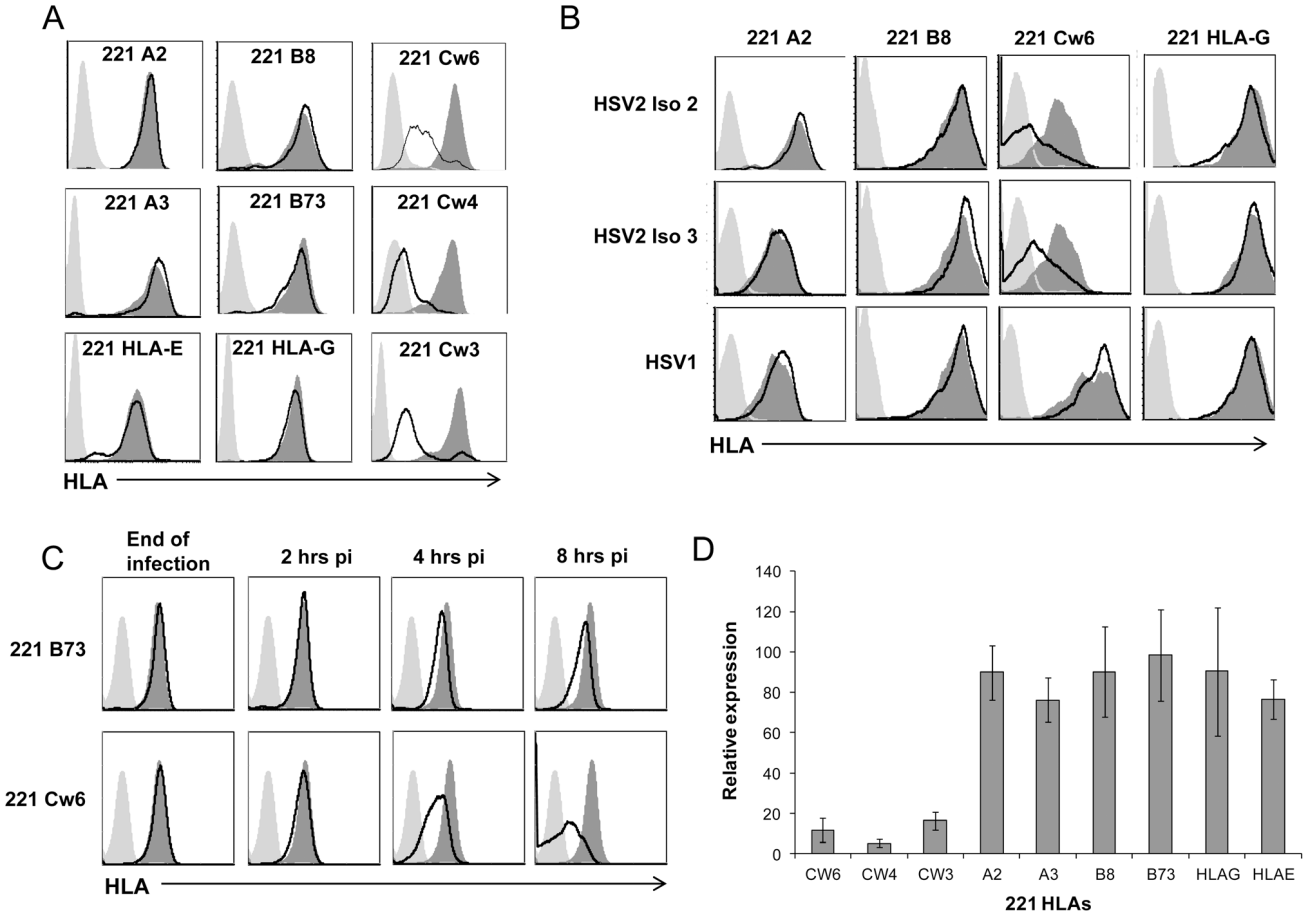
HSV-2 specifically down regulates HLA-C expression. (A) 721.221 (221) cells expressing various HLA proteins (indicated in the histograms) were infected with HSV-2 (MOI of 0.5) and the levels of the various HLA proteins expression were determined by FACS (black empty histograms represents the staining of infected cells). (B) Two clinical isolates of HSV-2 (HSV2 Iso 2 and HSV2 Iso 3) and one clinical isolate of HSV-1 were used to infect the various 721.221 transfectants indicated in the figures (MOI of 0.5 for all infections) and the levels of the HLA expression were measured FACS (black empty histograms represent the infected cell staining). (C) 221 expressing HLA-B73 or HLA-Cw6 cells were infected with HSV-2 (MOI of 1) and HLA expression was assessed right at the end of the infection, and at the indicated time points post infection (pi). In A–C the dark gray shaded histograms represent staining of the corresponding uninfected cells and the background levels (light grey shaded histogram) are the APC-conjugated Abs. For all panels, one representative experiment is shown out of at least three performed. (D) Quantification of all experiments performed in (A). The expression of each HLA allele without infection was set up to be 100%. Shown are relative average MFI ± S.D. from four to eight independent experiments.

The enhanced killing observed following HSV-2 infection might be explained either by a reduction in the expression of inhibitory ligands or by an up regulation of activating ligands. To investigate this we infected the HeLa cell line (since it expresses numerous NK activating ligands) with HSV-2 and monitored the expression of various NK cell ligands, at different time points following infection. The infection was performed with low MOI of 0.1 and therefore expression of the various ligands could be examined over relatively long period of time (144 hours, Figure 4) until the cells had died due to the infection. As can be seen in figure 4, 48 hours following HSV-2 infection we observed some reduction in the expression of several NK ligands, but this reduction was not consistent as it was not observed 144 hours post infection. Therefore we did not consider this reduction meaningful. The only consistent change we observed over time was the reduction in the expression of MHC-I proteins (Figure 4). Similar results were observed when HFF cells were used (data not shown). To examine whether the HSV-2-mediated MHC-I down regulation is a general mechanism or whether it affects only specific MHC-I alleles we have used the class I negative cell line 721.221 (221) and 721.221 cells that were transfected with various MHC-I proteins: two HLA-A alleles -A2 and -A3, two B alleles -B8 and -B73, three HLA-C alleles -Cw3, -Cw4 and -Cw6 and two non-classical MHC-I proteins HLA-E and HLA-G (Figure 5A). The various transfectants were infected with HSV-2 (MOI of 0.5) and the levels of the various HLA class I proteins were evaluated. Remarkably, a marked reduction of HLA-C expression was observed following HSV-2 infection, whereas little or no reduction in the expression levels of other MHC proteins was noted (Figure 5A). No reduction of HLA-C expression was detected when a UV-inactivated virus was used (data not shown). The specific reduction of HLA-C expression was not restricted to a certain HSV-2 virus strain, as infection with 2 other clinical isolates of HSV-2 gave similar results (Figure 5B). Furthermore, although HSV-1 and HSV-2 are approximately 50% homologous [25], the specific down regulation of HLA-C was not observed following HSV-1 infection (Figure 5B). We next tested the kinetics of HLA-C down regulation and observed a substantial reduction in HLA-C expression as early as 4 hours post infection (Figure 5C). Sometimes we also observed a slight reduction in other MHC-I alleles such as HLA-B73. However, this reduction was not consistent and as can be seen in figure 5D (which summarizes several FACS experiments performed with infected 721.221 transfectants), the HSV-2-mediated reduction of MHC-I proteins other than HLA-C was minimal.

### Abolishment of NK Cell Inhibition

**Figure 6:**
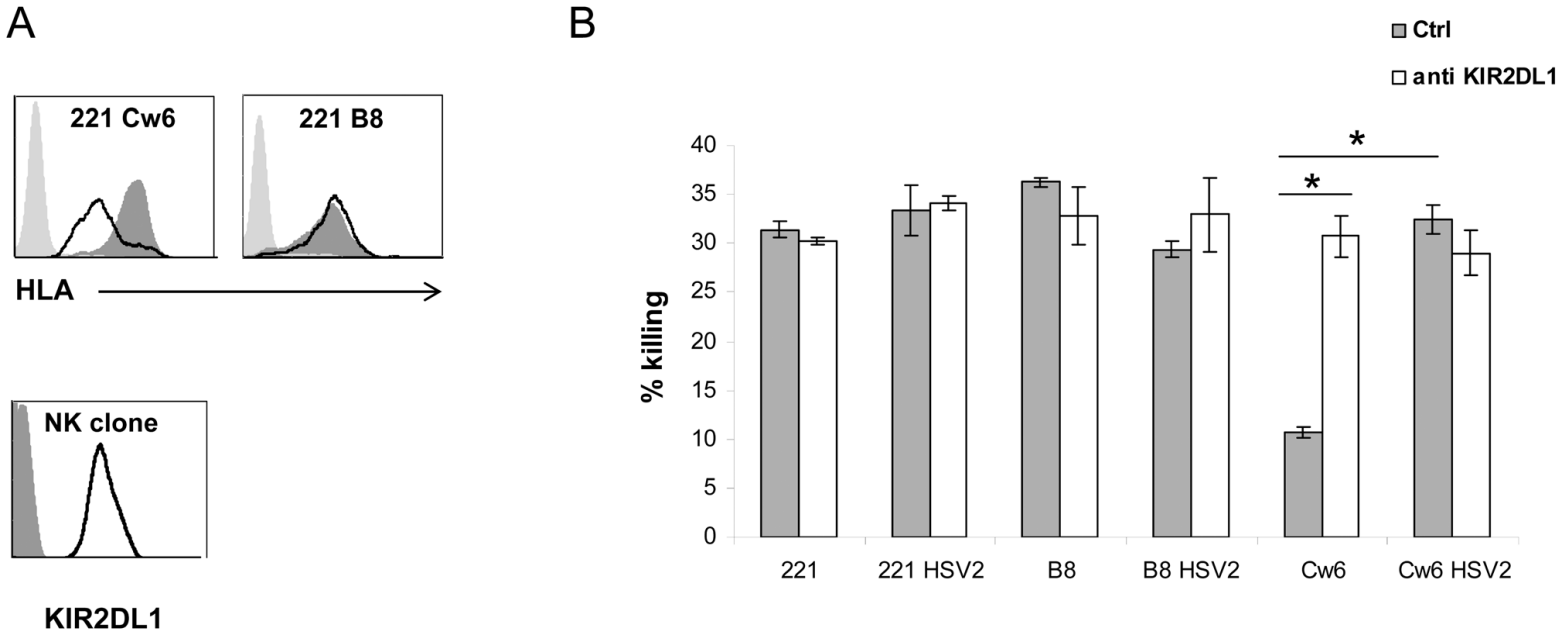
The KIR2DL1-mediated inhibition is abolished in HSV-2 infected cells. (A) Top histograms: 221 cells expressing either HLA-B8 or HLA-Cw6 were infected with HSV-2 (MOI of 0.5). The levels of HLAs expression were determined by FACS (black empty histograms represent infected cells). The dark gray shaded histograms represent staining of the corresponding uninfected cells. Background levels (light grey shaded histogram) are the APC-conjugated Abs. Lower histogram: Expression of KIR2DL1 (black empty histograms) was detected with anti-KIR2DL1 antibody. Background levels (grey shaded histogram) are the APC-conjugated Abs. (B) Killing assay. 221 and 221 expressing either HLA-B8 or HLA-Cw6 were infected with HSV-2 (MOI of 0.5). Uninfected and infected (indicated in the figure as HSV2) 221 cells were radioactively labeled and incubated with primary KIR2DL1-positive NK clones for 5 hours at an E:T ratio of 10∶1. The killing assays were performed 48 hours post infection and the NK clone was incubated either with a control mAb (Ctrl, grey columns) or with an anti-KIR2DL1 blocking antibody (white columns). Shown are mean values ± SD. Statistically significant differences are indicated (_*_ p≤0.0005, by one-tailed t test). Error bars (SD) are derived from triplicates. For all panels, one representative experiment is shown out of at least three performed.

To test if the reduction in HLA-C expression will affect NK cell inhibition we infected the parental 221 cells, 221 cells expressing B8 and 221 cells expressing Cw6 with HSV-2 and verified that the infection lead, as above, to specific down regulation of HLA-C (Figure 6A). Infected and non-infected cells were next subjected to killing by KIR2DL1-positive NK clones (example is shown in Figure 6A, bottom) as this receptor recognizes HLA-Cw6 [10], [26]. As expected, inhibition of NK cytotoxicity was observed only with uninfected cells expressing HLA-Cw6 (Figure 6B). This inhibition resulted from the interaction between KIR2DL1 and Cw6 as it was abrogated following blockage by anti-KIR2DL1 antibody (Figure 6B). Importantly, upon HSV-2 infection the inhibition was lost and the infected Cw6 expressing cells were killed as efficiently as all other cells (Figure 6B). Similar results were obtained with additional KIR2DL1-positive NK clones (data not shown).

### Glutamic Acid in the Tail of HLA-C Is Targeted by HSV-2

**Figure 7:**
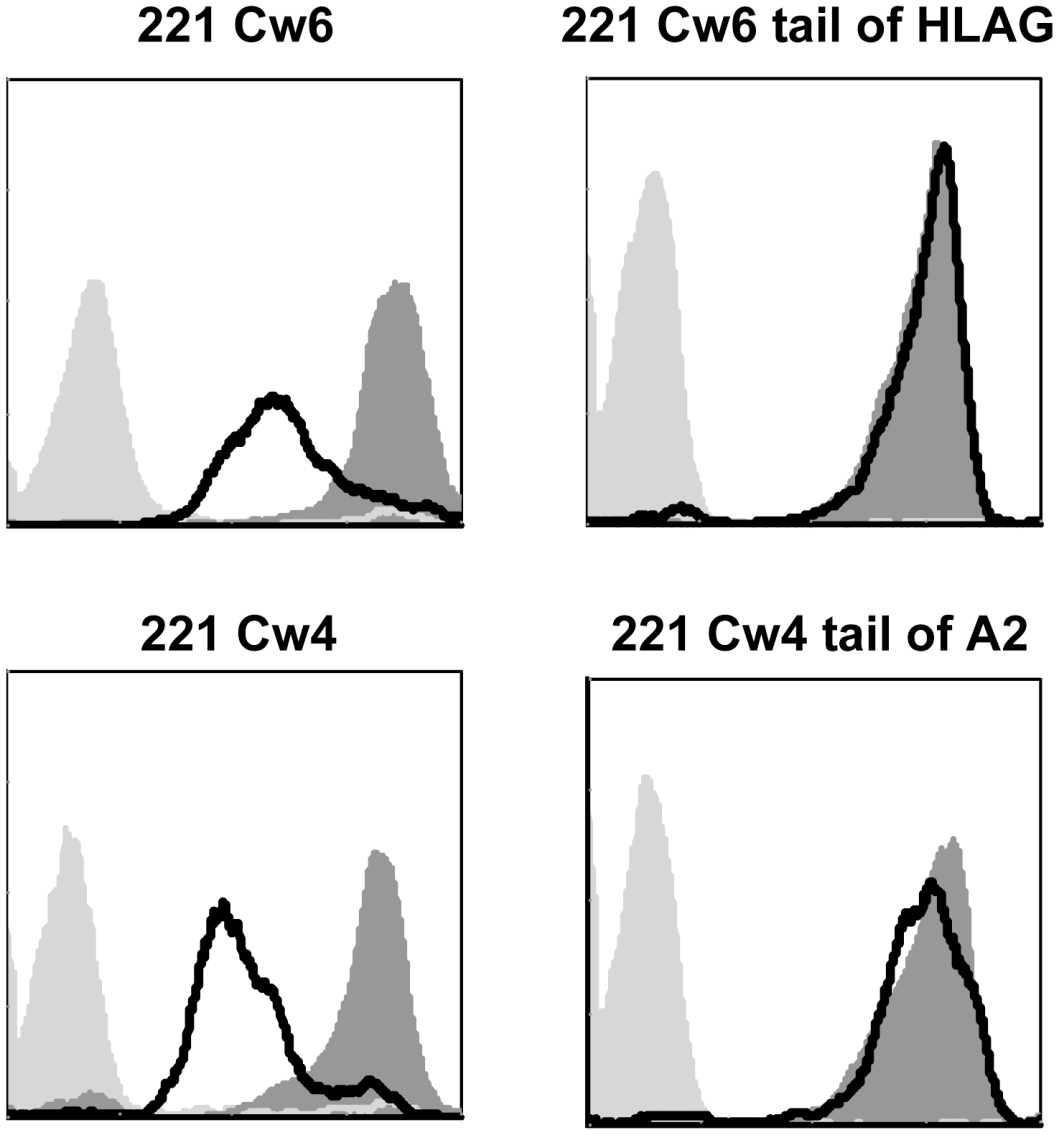
HSV-2 targets the cytoplasmic tail of HLA-C. 221 cells expressing HLA-Cw4, HLA-Cw6, HLA-Cw4 with the cytoplasmic tail of HLA-A2 and HLA-Cw6 with the cytoplasmic tail of HLA-G (indicated above the histograms) were infected with HSV-2 (MOI of 0.5). The levels of the various HLAs were determined by FACS (black empty histograms represents the staining of the infected cells). The dark gray shaded histograms represent staining of the corresponding uninfected cells. Background levels (light grey shaded histogram) are the APC-conjugated Abs. Figure show one representative experiment out of four performed.

**Figure 8:**
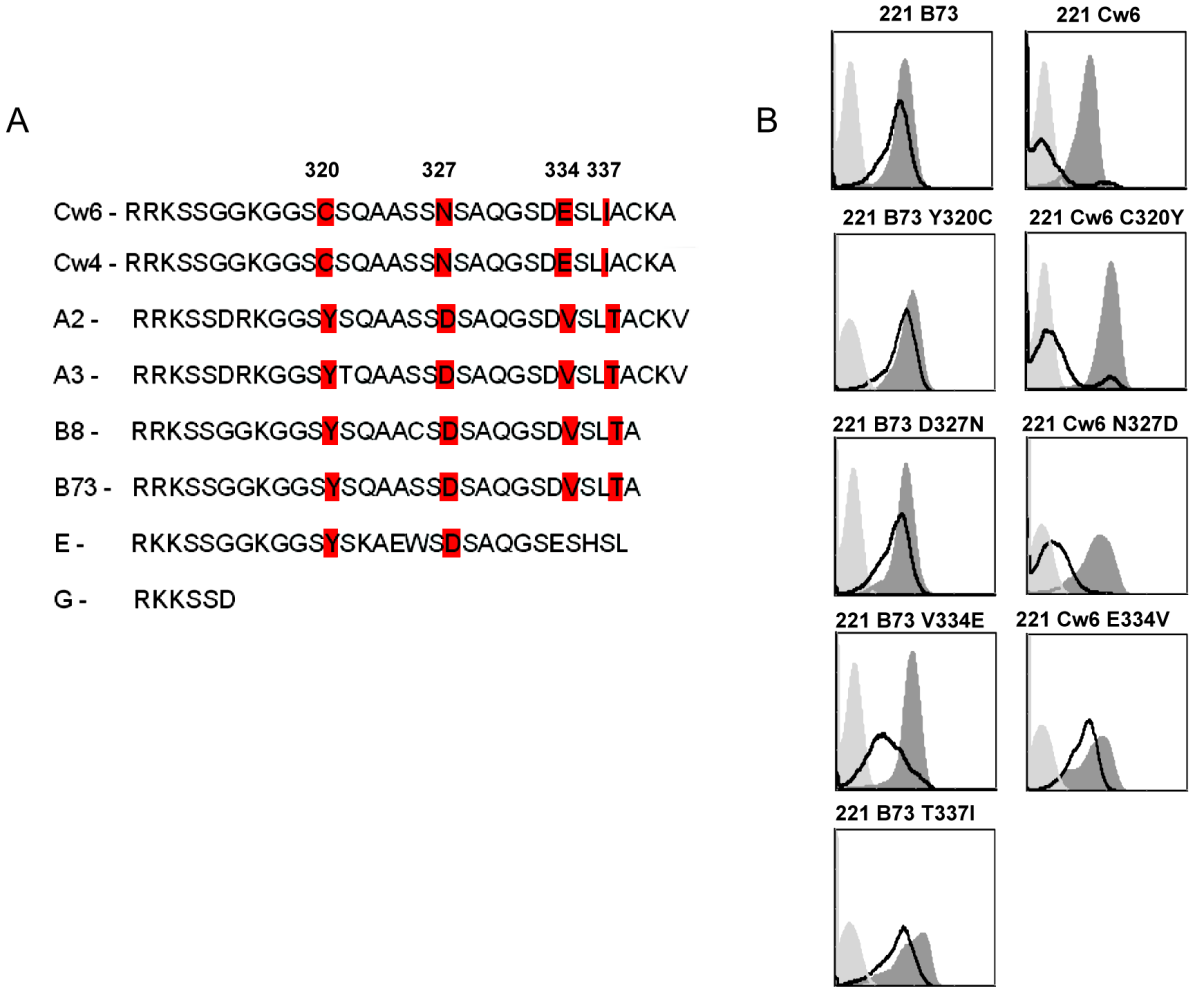
The downregulation of HLA-Cw6 by HSV-2 is dependent on the glutamic acid residue located in position 334. (A) Schematic representation of the cytoplasmic tails of various HLAs. Highlighted in red are the amino acid that are different between HLA-C and other HLA proteins. (B) The various 221 cells (expressing either wild type or mutants HLA proteins, indicated on top of the histograms) were infected with HSV-2 (MOI of 0.5). The levels of the various HLAs was determined by FACS (black empty histograms represent the infected cell staining). The dark gray shaded histograms represent staining of the corresponding uninfected cells. Background levels (light grey shaded histogram) are the APC-conjugated Abs. Figure show one representative experiment out of 3 performed.

To elucidate the mechanism leading to the HSV-2-mediated down regulation of HLA-C we concentrated on the cytoplasmic tail of HLA-C molecules. This is because a similar, yet opposite, tail-dependent mechanism is used by the HIV Nef protein to down regulate HLA-A and HLA-B molecules and not HLA-C [14]. We replaced the tail of HLA-Cw4 with the tail of HLA-A2 and the tail of HLA-Cw6 with the tail of HLA-G. The various proteins were expressed in 221 cells resulting in the generation of the following transfectants: 221 Cw4, 221 Cw6, 221 Cw4 tail of A2 and 221 Cw6 tail of HLA-G. All transfectants were next infected with HSV-2 and the expression levels of the various HLA class I proteins were evaluated. As can be seen in figure 7, HSV-2 indeed specifically targets the tail of HLA-C because when it was replaced, either with the tail of A2 or with the HLA-G tail, the down regulation of HLA-C proteins (either Cw4 or Cw6) was abolished. To identify the HLA-C residue/s that are involved in the HSV-2-mediated HLA-C down regulation we searched for residues which are found in the tail of HLA-C alleles and absent in other HLA class I molecules. Four such residues were found: C320, N327, E334 and I337 (Figure 8A). To study which of the four amino acids is important for the HSV-2-mediated HLA-C down regulation, we inserted point mutation(s) in each of the HLA-C-specific amino acid residues of HLA-Cw6 converting them into the corresponding amino acids that are present in the cytoplasmic tail of HLA-B73 and other B alleles (Cw6 C320Y, Cw6 N327D, Cw6 E334V, for some unknown reason expression of the Cw6 I337T mutation was not detected on the cell surface). The reciprocal mutations in which the HLA-C residues were inserted into the B73 proteins were also performed (B73 Y320C, B73 D327N, B73 V334E and B73 T337I). All 7 proteins were expressed in 721.221 cells and the expression of the corresponding molecules was evaluated before and after HSV-2 infection. Importantly, we discovered that residue 334 (E in Cw6) is the primary residue involved in the HSV-2-mediated HLA-C down regulation. This is because the Cw6 down regulation was almost completely abolished when this residue was mutated (Figure 8B). And vice versa, mutating the B73 residue in position 334 from V to E resulted in efficient down regulation of B73 following HSV-2 infection (Figure 8B). As above, although sometimes, little down regulation of HLA-B was also observed following HSV-2 infection (Figure 8B), this down regulation was much weaker as compared to Cw6 and the most efficient down regulation of B73 was observed only when its V residue at position 334 was replaced with E. Thus, glutamic acid in position 334 of Cw6 is targeted by HSV-2.

### The ICP47 Protein of HSV-2 Mediates the Specific HLA-C Down Regulation

**Figure 9:**
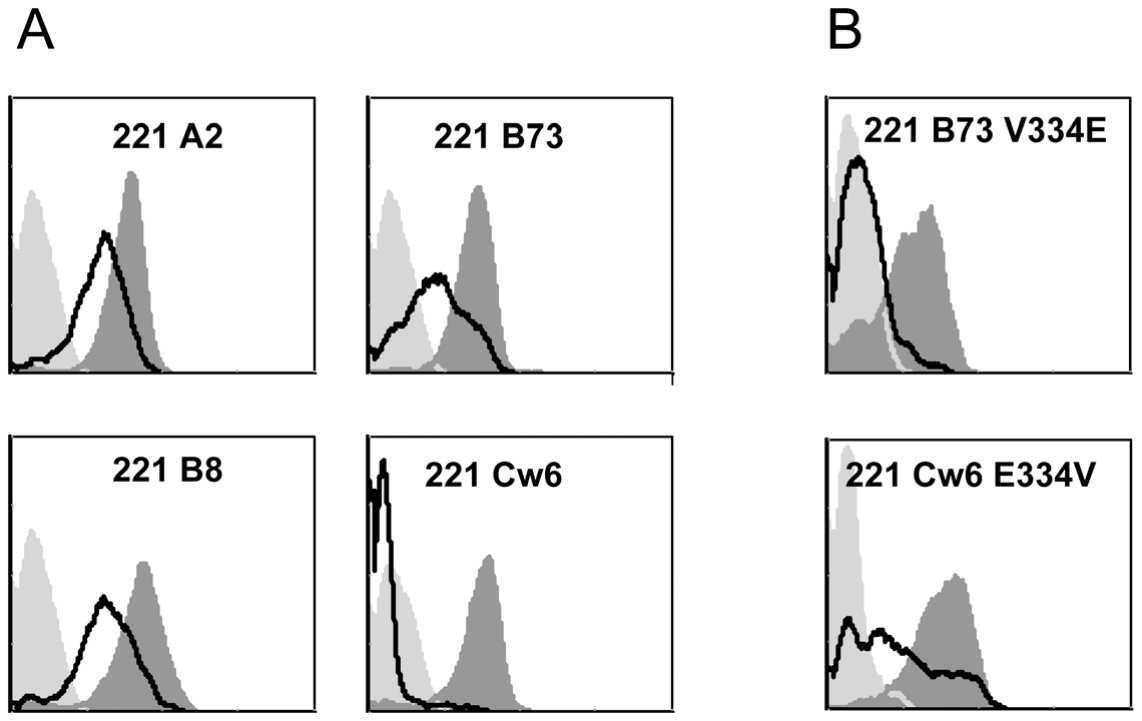
The ICP47 protein of HSV-2 reduces HLA-C expression. Various 221 cells expressing wild type (A) and mutants (B) HLA proteins (indicated in the figure) were transduced with lenti virus vectors expressing ICP47 and GFP (indicative for the transfection efficiency). The levels of the various HLAs were determined by FACS (black empty histograms represent the ICP47 transduced cells). The dark gray shaded histograms represent staining of the corresponding cells transduced with a control lenti virus vector. Background levels (light grey shaded histogram) are the APC-conjugated Abs. Figure show one representative experiment out of 3 performed.

It is well establish that the ICP47 proteins of HSV-2 inhibit TAP in epithelial cells and that this consequently leads to HLA down regulation [22]. However, it is also known that APCs are not sensitive to the ICP47-mediated TAP inhibition. Thus, it seemed to us unlikely that the virus will develop a protein the inefficiently inhibits TAP in APCs and we were wondering whether ICP47 has additional functions which are TAP-independent. Furthermore, it is known that often different immune evasion mechanisms are utilized by the same viral protein [27]–[29]. Therefore, we hypothesized that ICP47 might be responsible for the specific HLA-C down regulation that occurs following HSV-2 infection. To investigate this, we cloned the ICP47 cDNA into a lenti virus vector that also contains GFP and transduced 221 cells and various 221 transfectants expressing various MHC-I proteins. The transduction efficiency (as determined by the GFP expression) was high (data not shown) and the analysis was performed on the GFP-positive cells. As can be seen in figure 9A when ICP47 was expressed in 721.221 cells expressing HLA-A2, HLA-B73 or HLA-B8 a modest down regulation was observed. Importantly, the expression of HLA-C was completely abolished following the ICP47 transduction (Figure 9A). To test whether this complete ICP47-dependent reduction of Cw6 is dependent on the E334 residue we transduced 221 cells expressing either Cw6 E334V or B73 V334E with lenti viruses expressing ICP47. The transduction efficiency was very high (data not shown) and the analysis was performed on the GFP-positive cells. As can be seen in figure 9B, the complete ICP47-mediated reduction that was observed in 221 Cw6 cells was not observed the 221 Cw6 E334V cells and some expression of Cw6 E334V was maintained on the cell surface even in the presence of ICP47 (compare figures 9A and B). The reciprocal picture was seen in the transduced 221 HLA-B73 cells. When ICP47 was expressed in 221 B73 V334E cells a complete down regulation was observed in contrast to 221 HLA-B73 cells in which only partial down regulation was detected (compare figures 9A and B). Thus, the ICP47 protein of HSV-2 down regulates HLA expression using two different mechanisms: (i) through TAP inhibition (a general, inefficient mechanism that function primarily in non-APCs) and (ii) through its interaction with glutamic acid residue present in position 334 of the cytoplasmic tail of HLA-C (a specific HLA-C-dependent mechanism).

### HSV-2 Infection of DC Resulted in Reduced HLA-C Expression and Increased NK Cell Cytotoxicity

**Figure 10:**
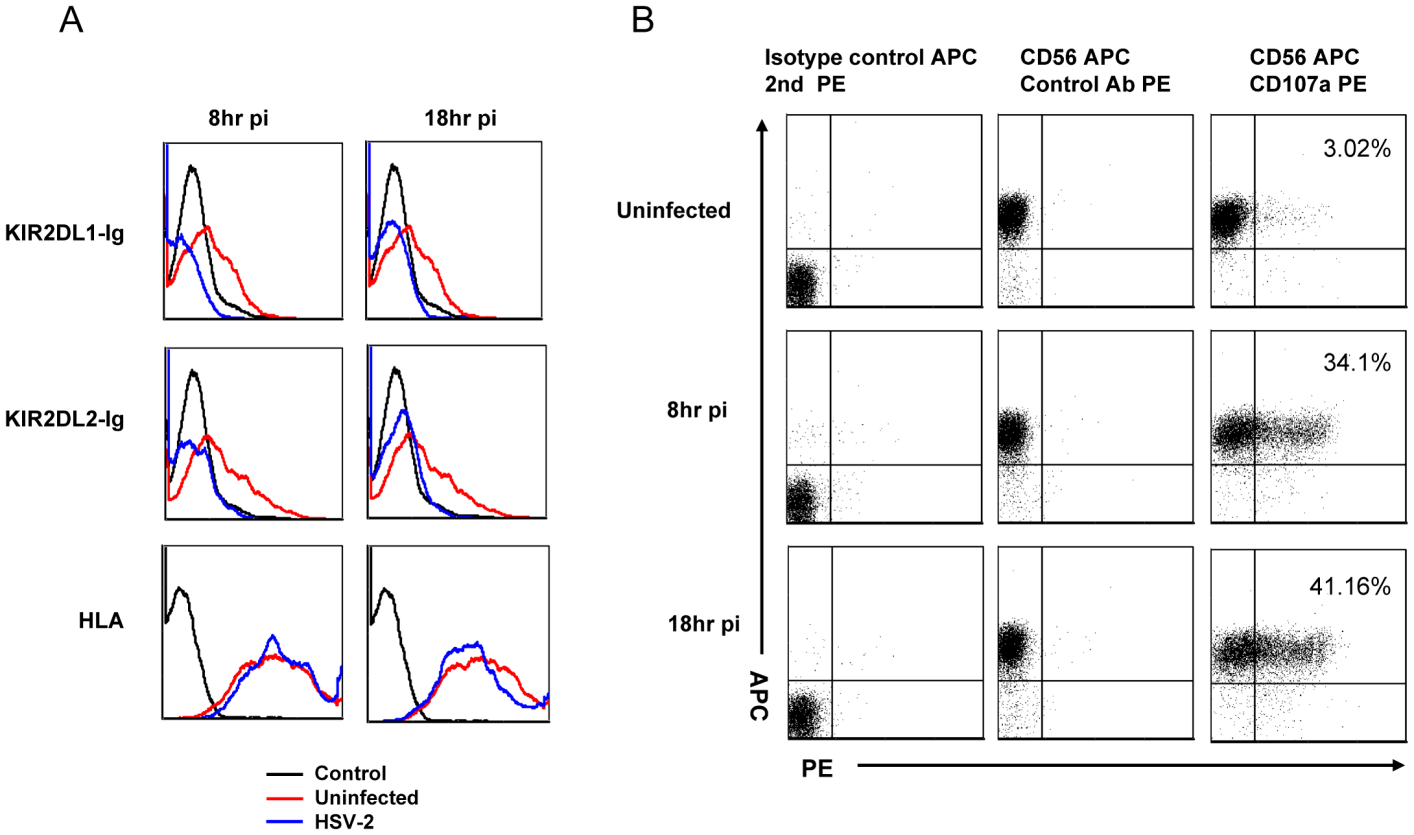
Infection of DCs by HSV-2 leads to HLA-C down regulation and consequently enhance NK cell killing. (A) DCs were infected with HSV-2 (MOI of 0.5), staining was performed 8 and 18 hours post infection (pi). The levels of HLA expression were determined by FACS (blue histograms, represent infected cells, red histograms represent uninfected cells). Top histograms: binding of KIR2DLI-Ig, middle histograms: binding of KIR2DL2-Ig, lower histograms; binding of the pan anti-MHC-I W6/32 antibody. Background levels (black empty histogram) are the APC-conjugated Abs. (B) Uninfected and HSV-2 infected DCs were incubated for 2 hours with primary bulk NK cells, 8 or for 18 hours post infection. NK cells were identified by staining for the expression of CD56 and the levels of NK cell degranulation was monitored by anti CD107a mAb. The percentages of NK cell degranulation are indicated. The staining was quantified by FACS. Figure show one representative experiment out of two performed.

We next wonder why a virus would develop a mechanism (specific down regulation of HLA-C) that enables a better killing of the infected cells. Our assumption was that HSV-2 induces a selective down regulation of HLA-C proteins, primarily in APCs, to interfere with the activation of the adaptive immunity. Indeed it is known that DCs express the HSV-2 entry receptors, HVEM and nectin-2 and are therefore highly susceptible to HSV infection [30]. Hence we decided to test whether HSV-2 infection of DC will result in specific HLA-C downregulation and consequently increased NK cell killing. Testing for selective down regulation of HLA-C on DC is difficult due to the lack of HLA-C specific mAbs. Therefore, to overcome this problem, we used fusion proteins composed from the extra cellular portions of KIR2DL1 and KIR2DL2 (that recognizes specifically the entire spectrum of HLA-C proteins [8]) fused to human IgG1 (KIR2DL1-Ig and KIR2DL2-Ig, respectively). The general expression levels of MHC-I proteins were determined by using the pan anti-MHC-I mAb W6/32. As can be seen in figure 10A, HSV-2 infection of DC resulted in reduced expression of HLA-C, as it abolished the binding of both KIR2DL1-Ig and KIR2DL2-Ig fusion proteins. This specific reduction was observed as early as 8 hours following HSV-2 infection. In contrast, little or no reduction in the general expression levels of MHC-I proteins was observed. This is probably because the levels of HLA-C is only around 10% of that of HLA-A and HLA-B proteins [31]. Similar results were obtained with other DCs derived from additional donors (data not shown). Next, we tested whether the down regulation of HLA-C will affect the NK cell mediate killing. For that we incubated primary bulk NK cell cultures with HSV-2-infected and uninfected DCs and stained the NK cells for the expression of CD107a (LAMP-1, a marker for cytoplasmic granules release and NK cell degranulation). As can be seen, following DC infection, 34% and 41% of the NK cells degranulated (as evidence from the CD107a staining), 8 and 18 hours post infection respectively, while incubation with uninfected DCs led to only 3% degranulation (Figure 10B).

## Discussion

NK cells and viruses co-evolved for millions of years. This co-existence led to the development of opposite mechanisms in which the NK cells try to kill the infected cells and the viruses on the other hand try to escape such killing [32]. Famous among these viruses are viruses of the Herpesviride that had developed numerous immune evasion mechanisms such as the use of viral miRNAs in order to escape NK cell attack [23], [24], [33]. Hence, we were quite surprised to observe that no changes were detected in the expression of the NK ligands tested, after transduction with 21 miRNAs of HSV-2 and that increased NK cell killing was observed following HSV-2 infection. Indeed it was shown that NK cells play an important role in controlling HSV-2 infections. It was previously shown that IL-15^−/−^ mice, which lack NK and NKT cells, were more susceptible to HSV-2 infection than mice lacking both T and B cells [18]. In addition, it was demonstrated that CCR5 deficient mice suffer from increased susceptibility to genital challenge with HSV-2. The absence of CCR5 had no significant impact on T-cell mobilization or recruitment to sites of infection, rather, NK cell infiltration was diminished, and a reduction in NK cell activity was observed [34]. Finally, it was recently shown that human NK cells contributed significantly to the stimulation of CD4 T lymphocytes through direct presentation of HSV1/2 antigens to CD4 T lymphocytes [35]. To understand the reasons accounting for the increased killing of HSV-2 infected cells, we evaluated the expression of various NK ligands following infection and observed reduction in the expression levels of MHC-I proteins in infected cells. This finding was quite expected as it was shown that HSV-2 encodes for the ICP47 protein that binds TAP and therefore leads to a general MHC-I down regulation [36]. However, ICP47 does not efficiently down regulate MHC-I in cells expressing high levels of TAP such as B cells and DCs [22]. Indeed we observed, when using the EBV transformed B cell line 721.221 and BM-derived human DCs that HSV-2 infection leads to an allele-specific down regulation of HLA-C, which consequently rendered the infected 721.221 cells and DC susceptible to NK cell killing. Moreover, we showed that HSV-2 through its ICP47 protein down regulates HLA-C by targeting the glutamic acid residue present in position 334 of the cytoplasmic tail of HLA-C. An interesting question is why HLA-C contains this residue and why it is not mutated to avoid this virus tactic. One possible answer is that this residue also regulates the expression of HLA-C under normal conditions and therefore it is indispensible. Indeed, HLA-C is expressed in lower levels as compared with HLA-A and HLA-B [31] and it was shown that the surface expression of HLA-C is regulated through multiple mechanisms, some of which are unknown [37]. Cellular pathways/proteins that are important for immune evasion of herpes viruses are often targeted by several viral proteins and sometimes even by viral proteins combined with viral miRNAs, as we have shown regarding the NKG2D ligands [23], [24], [38]. With regard to MHC-I it was shown, for example, that HCMV encodes at least four proteins (US2, US3, US6 and US11) that function to reduce MHC-I expression. HCMV also encodes for the UL18 protein that binds the inhibitory receptor ILT-2 and for the UL40 protein that provides a leader peptide, which is presented by HLA-E [15]. Furthermore, occasionally a single viral protein possesses multiple immune evasion properties [27]–[29]. For example, the latent membrane protein 1 (LMP1) of Epstein-Barr virus (EBV) mimics CD40 signaling, functions as a viral oncogene, affects cell-cell contact, cytokine and chemokine production and antigen presentation [29]. Another example is the K5 protein that functions as E3 ligase and degrades several immune-related ligands [27], [39]. Thus we hypothesized that the ICP47 protein which was shown previously to affect MHC-I expression in non-APC is responsible for the specific HLA-C down regulation observed in APC. Importantly we demonstrate that when ICP47 was expressed in cells expressing Cw6 a complete down regulation was observed and when the E334 residue of Cw6 was converted to V the expression of Cw6 E334V was detected on the cell surface even in the presence of ICP47. The reciprocal picture was seen with regard to HLA-B73. The expression of Cw6 E334V in the presence of ICP47 was not completely restored probably because ICP47 still function as TAP inhibitor in these B cells (as it expressed in high levels). In addition, mutating the E334 residue into V did not completely restored the expression of Cw6 in cells infected with HSV-2, suggesting that additional elements in the tail of Cw6 might be involved in the ICP47-mediated down regulation. We therefore propose that ICP47 affects HLA expression by using two different mechanisms 1) Via inhibition of the TAP transporter which leads to a general down regulation of various HLA-I proteins especially in cells that express low levels of TAP and 2) Via a specific down regulation of HLA-C molecules, a mechanism that is primarily dependent on the E334 residue located in the HLA-C tail that occurs in cells expressing high levels of TAP such as APC. It was shown that immature DCs express unknown ligands for the NK killer receptors NKp46 and NKp30 and that they are killed by NK cells, while mature DCs are protected from killing because they express high levels of MHC-I proteins [40]. We demonstrated here that the down regulation of HLA-C following DC infection with HSV-2 renders them susceptible to NK cell killing. We therefore suggest that in the infected tissue the virus uses ICP47 to avoid CTL attack and in DCs it uses ICP47 to specifically target HLA-C and consequently to render DCs susceptible to NK cell elimination. By killing DCs which are central activators of T helper cells and CTLs, through cross presentation, HSV-2 avoids adaptive immune responses. Interestingly, a similar example was described before. The ICP10 protein of HSV-2 induces apoptosis in immune cells, while its expression protects epithelial cells from apoptosis [41].

HSV-2 is a human pathogen that co-evolved with its human host for millions of years. Thus, the virus had developed mechanisms to avoid specifically the human immune response. ICP47, for example, blocks TAP in human fibroblasts, however, almost no inhibition of the mouse TAP is observed in a variety of mouse cells, unless ICP47 is applied in high concentrations (50 to 100-fold higher than those required to inhibit the human TAP) [21], [22]. More importantly, the new mechanism that we have discovered here in which the virus target HLA-C proteins is unique to humans as HLA-C is not expressed in the mouse. This novel human-specific immune evasion mechanism in which the virus harness NK cells for its own benefit demonstrates yet again the sophistication of herpesviruses in general and of HSV-2 in particular.

## Materials and Methods

### Ethics Statement

The NK cells that were used in this study were obtained from the blood of healthy voluntaries. The intuitional Helsinki committee of Hadassah approved the study (Helsinki number 0030-12-HMO). All subjects provided a written informed consent.

### Lentiviral Constructs and Transduction

RNA artificial hairpins that function as orthologs of pre-miRNA hairpins were generated by using the pTER vector. Two complementary specific oligonucleotides were annealed, phosphorylated using T4 polynucleotide kinase as was previously described [42] and inserted into the pTER vector. The artificial hairpin and H1 RNA polymerase III promoter were excised from the vector and cloned into the lentiviral vector SIN18- pRLL-hEFIap-EGFP-WRPE [43]. The lentiviral vector contains GFP, thus allowing the simultaneous expression of both reporter GFP and miRNA. Lentiviral viruses were produced by transient three-plasmid transfection as described [43].

### FACS Staining, Antibodies and Fusion Proteins

The KIR2DL1-Ig and KIR2DL2-Ig fusion proteins were generated in COS-7 cells and purified by affinity chromatography using a protein G column, as described previously [44]. The various 721.221 expressing HLA proteins were stained using W6/32 mAb directed against MHC-I, anti human HLA-E mAb (clone MEM-E07, Serotec) and anti human HLA-G mAb (clone MEM-G9, Serotec). Anti-MICA, anti-MICB, anti-ULBP1, anti-ULBP2, anti-ULBP3, anti- ICAM1 and anti-PVR antibodies were all purchased from R&D Systems (Minneapolis).The staining of cell lines was visualized using a secondary Allophycocyanin (APC) conjugated goat anti-human Abs (Jackson ImmunoResearch Laboratories, West Grove, PA) and a secondary APC conjugated goat anti-mouse Abs (Jackson ImmunoResearch Laboratories, West Grove, PA).

### Cells and Virus Infection

The 721.221, RKO, DU145, HeLa, Hep3b, 293T and MCF7 cell lines were used. 721.221 transfectants (A2, A3, B8, B73, Cw3, Cw4, Cw6, HLA-E,HLA-G, Cw4 tail of A2 and Cw6 tail of HLA-G) were generated previously as described [14]. Infection with three HSV-2 clinical isolations and HSV-1 17+/pR20 5/5 strain was done at a multiplicity of infection (MOI) as described in each experiment. Primary NK cells from healthy donors were isolated from PBLs using the human NK cell isolation kit and the autoMACS instrument (Miltenyi Biotec, Auburn, CA) according to the manufacturer's instruction. They were grown as described [10]. KIR2DL1 positive NK clones were identified by flow cytometry using the anti-KIR2DL1 mAb HP3E4.

### Generation of 721.221 Cells Expressing Mutated HLA Class I Proteins

For mutating the MHC-I proteins, we amplified two overlapping fragments of the gene by PCR. The upstream fragment was amplified using a gene-specific 5′ primer (including the BamHI restriction site) and an internal 3′ primer that contains the mutation. The downstream fragment was amplified using an internal 5′ primer containing the mutation and a gene-specific 3′ primer (including Bsp1407I restriction site). Next, both purified fragments were mixed together with the 5′ primer and the 3′ primer to generate the mutated full-gene cDNA. Mutations in the end of the 3′ were performed by using the gene-specific 5′ primer and 3′ primer that includes the mutation. The various cDNAs were cloned into the lentiviral vector SIN18-pRLL-hEFIp-EGFP-WRPE by removing the GFP gene and inserting the HLA gene and used to infect 721.221 cells. Lentiviral viruses were produced by transient three-plasmid transfection as described [43]. These viruses were used to transduce 721.221 cells in the presence of polybrene (5 g/ml).

### Cytotoxicity Assay

The cytotoxic activity of NK cells against target cells was assessed in 5-hour 35S-release assays, as described [45] E:T ratio was 10∶1. The final concentration of the blocking antibodies (anti-KIR2DL1 mAb HP3E4) was 2.5 µg/ml.

### CD107a Assays

NK cells (5×10^5^) were co-incubated with DCs in a ratio of 1∶1 in the presence of 0.2 µg of a Bioten-conjugated CD107a Ab (1D4B; Southern Biotechnology Associates, Birmingham, AL) for 2 h. Bioten-conjugated CD153 Ab was used as control, the staining was visualized using a secondary R-Phycoerythrin (PE)-conjugated streptavidin (Jackson ImmunoResearch Laboratories, West Grove, PA). NK cells were identified by APC-conjugated CD56 mAb (HCD56; Biolegend, San Diego, CA). Afterward; cells were washed and analyzed by FACS.

### Cloning and Expression of ICP47 Protein

The ICP47 the gene was amplified by PCR from cDNA of 221 HSV-2 infected cells. The cDNA was cloned into the lentiviral vector DsRed (−) wich also includes a reporter GFP. Lentiviral viruses were produced by transient three-plasmid transfection as described [43] these viruses were used to transduce 721.221 cells in the presence of polybrene (5 g/ml).
